# Autophagy and the (Pro)renin Receptor

**DOI:** 10.3389/fendo.2013.00155

**Published:** 2013-10-21

**Authors:** Katrina J. Binger, Dominik N. Muller

**Affiliations:** ^1^Experimental and Clinical Research Centre, Max Delbrück Center for Molecular Medicine, Berlin, Germany

**Keywords:** renin-angiotensin system, cardiovascular disease, Wnt signaling, vacuolar H^+^-ATPase, proteostasis, autophagosome, rapamycin

## Abstract

The (pro)renin receptor (PRR) is a newly reported member of the renin-angiotensin system (RAS); a hormonal cascade responsible for regulating blood pressure. Originally, identification of PRR was heralded as the next drug target of the RAS, of which such therapies would have increased benefits against target-organ damage and hypertension. However, in the years since its discovery, several conditional knockout mouse models of PRR have demonstrated an essential role for this receptor unrelated to the RAS and blood pressure. Specific deletion of PRR in podocytes or cardiomyocytes resulted in the rapid onset of organ failure and subsequently animal mortality after only a matter of weeks. In both cell types, loss of PRR resulted in the intracellular accumulation of autophagosomes and misfolded proteins, indicating a disturbance in autophagy. In light of the fact that the majority of PRR is located intracellularly, this molecular function appears to be more relevant than its ability to bind to high, non-physiological concentrations of (pro)renin. This review will focus on the role of PRR in autophagy and its importance in maintaining cellular homeostasis. Understanding the link between PRR, autophagy and how its loss results in cell death will be essential for deciphering its role in physiology and pathology.

## PRR and the Renin-Angiotensin System

The discovery of the (pro)renin receptor (PRR) by Ngyuen et al. (1) came at a time in the field of hypertension and cardiovascular disease when the search was on for a new therapeutic target of the renin-angiotensin-system (RAS). Normally, this hormone system is responsible for the regulation of blood pressure and water retention. Under pathological conditions, overactivity of the RAS results in the downstream accumulation of angiotensin (ang) II, an octapeptide which binds to specific receptors in target organs such as the kidney and heart. Pathologically high levels of ang II leads to hypertension and injury to target organs, resulting in disease (2). Numerous therapeutic interventions currently in use considerably reduce levels of ang II and are effective at lowering blood pressure (3). However, these drugs are not completely protective against target-organ damage, and as such, there is continued interest in the development of therapeutics against other components of the RAS which may have increased protection against disease.

The RAS is classically initiated by the proteolytic removal of the prosegment of prorenin to form active renin. Renin then starts the RAS cascade by cleaving angiotensinogen to form the decapeptide angiotensin I, which is further cleaved by the angiotensin-converting enzyme to form ang II. Upon discovery of PRR, an additional non-classical activation of prorenin was identified. Here, upon binding PRR, a conformational change in prorenin is thought to be induced, resulting in the removal of the prorenin prosegment from the active enzymatic cleft and hence, the non-proteolytic activation of prorenin into renin and initiation of the RAS cascade (1). This was the first study to show a molecular function for PRR and demonstrate its putative role in activation of the RAS. It should be noted however that the concentrations of prorenin used to observe this effect are considerably higher than those levels detected *in vivo* (4).

In addition to the activation of the RAS and generation of ang II, an alternative mechanism was proposed by which (pro)renin binding to the PRR directly contributes to disease. Human mesangial cells treated *in vitro* with recombinant human or rat renin showed an increase in transforming growth factor (TGF)-β and plasminogen activator inhibitor (PAI)-1 levels. In the presence of an ang II receptor blocker (ARB), the increase of TGF-β and PAI-1 was not affected, indicating that this result was independent of the RAS and ang II generation (5). In a human monocyte cell line, stimulation with recombinant renin in the presence of an ARB resulted in an activation of extracellular signal-related kinases (ERK) 1/2 (6). This again suggested that the increase in phospho-ERK 1/2 is directly due to the binding of prorenin to the PRR, and unrelated to the initiation of the RAS. Several other studies have also shown the activation of signaling pathways upon (pro)renin stimulation in an ang II independent manner (7–9). The identification of this second function for PRR led to the hypothesis that (pro)renin binding to the PRR causes pathology independently of the RAS *via* the induction of inflammatory and pro-fibrotic signaling cascades.

## A Murky Picture of PRR Function Emerges

### Therapeutic blockade of PRR prevents target-organ damage?

The aforementioned studies laid the framework for the development of inhibitors of PRR. To date, only one putative inhibitor of PRR has been published: the handle-region peptide (HRP). HRP is a short pentapeptide comprised from the prorenin prosegment (11P–15P). This putative inhibitor was developed by Suzuki et al. who screened antibodies raised against various epitopes of prorenin that would induce the non-proteolytic activation of prorenin (10). They identified two regions of prorenin, the “handle” (11P–15P) and “gate” (15P–26R) from which they deduced that these two regions are most important in the non-proteolytic activation of prorenin. The “handle” region was decided by the authors to be the most exposed epitope of prorenin and thus more likely to directly interact with PRR (10).

Administration of this peptide *in vivo* showed protection in several animal models of disease, specifically in diabetic microvascular complications (8, 11–13) and in a rat model of spontaneous hypertension (14). The authors did not identify a precise concentration at which HRP was most effective. Several other groups attempted to reproduce these studies however they were unable to show any efficacy (6, 15–17). A study by Wilkinson-Berka et al. developed a sensitive radioimmunoassay to detect plasma levels of HRP. These authors were not able to detect HRP in the plasma of SD rats infused with 1 mg/kg/day of HRP by mini-pump for 7 days, indicating the rapid metabolism of the peptide (13). This raises the question as to whether HRP is able to effectively traffic through the body to specifically inhibit PRR at the target-organ of interest.

It is important to note that HRP was developed at a time in which high-resolution structural information on prorenin was not known. Morales et al. have since solved the structure of prorenin and provide some explanation for the discrepancy between the various HRP studies (18). Based on their structural information, they show that the “handle” region is not an exposed epitope, as originally thought, but instead buried in the prorenin molecule. This is supported by a recent study which has shown that HRP does not specifically bind PRR with high affinity (19). This is in agreement with a study done by us, where we demonstrate non-specific binding of fluorescently labeled HRP to cells lacking PRR (15).

### Overexpression of PRR leads to hypertension?

As described above, the contribution of PRR to cardiovascular disease and hypertension was hypothesized to be due to the binding of prorenin to PRR, resulting in its non-proteolytic activation and/or the induction of signaling pathways which directly lead to pathology. The development of transgenic animal models over-expressing PRR were expected to shed more light on this, as with more PRR available it was hypothesized that more binding of (pro)renin would occur and thus more of these pathogenic processes would occur. Unfortunately, these animal models did not give a clear indication as to the molecular contribution of PRR to cardiovascular disease. Rats constitutively over-expressing human PRR had normal blood pressure and ang II levels but developed renal nephropathy (20). In contrast, in a different model in which transgenic rats overexpressed PRR solely in smooth muscle cells, these animals had elevated systolic blood pressure but normal renal function (21). The differing results from these two models have been proposed to be due to differences in the uncontrolled overexpression of PRR in these animals (22).

### Genetic ablation of PRR prevents hypertension and cardiovascular disease?

In consideration of the confusing results from the overexpression models and HRP studies, the field next turned to the development of knockout models to establish if loss of PRR would be protective against cardiovascular disease. The first attempt at generation of a PRR knockout mouse model was a failure. Injection of PRR knockout embryonic stem cells into host blastocysts did not generate chimeras, indicating an essential function of PRR in cellular development and survival (23). This is in contrast to other members of the RAS in which knockout mice have been successfully generated (24). Also of note is that renin expression begins well after PRR [in the mouse not until E14 (25)], again implying a non-RAS role for PRR. It is important to note here that when PRR was first discovered by Nguyen et al. it was shown to be identical to that of a protein called M8–9, a truncated protein of the vacuolar H^+^-ATPase (V-ATPase) (1). As the V-ATPase is a multifunctional protein essential for cellular homeostasis and development (26), these initial PRR knockout reports gave great support to the notion that PRR has functions separate to the RAS. Several other animal models have confirmed that loss of PRR has a profound effect on development, including zebrafish (27), *Xenopus* (28, 29), and *Drosophila* (28, 30). PRR also appears to be important for human development, as it has been identified that humans with mutations in PRR have intellectual disabilities and epilepsy (31). However, this singular study needs to be reassessed and validated in light of the information now available from whole genome sequencing (32).

Due to the developmental effects described for PRR, an alternative approach at understanding the role of PRR in cardiovascular disease was undertaken by Kinouchi et al. (33). Here, a conditional knockout model was generated in which PRR was specifically deleted in cardiomyocytes (ATP6AP2^lox/Y^; αMHC-Cre). These mice had a severe cardiac phenotype and died only 3 weeks after birth. Upon closer inspection of the PRR knockout cardiomyocytes, an accumulation of vesicular bodies was observed. Additionally, autophagosomes comprising of electron dense material were evident. By western blotting the authors show an accumulation of LC3B II and p62 in myocardial tissue from PRR knockout mice, indicating a disturbance in autophagic flux (33). In terms of the original question posed, this study did not give any insight as to how the binding of (pro)renin to PRR may contribute to cardiovascular disease. More recently, we and another group have generated podocyte-specific PRR knockout mice (ATP6AP2^lox/Y^; Podocin-Cre) (34, 35). In these two studies, knockout mice had again severe phenotypes and mortality after only 3 weeks. Similarly to PRR knockout in cardiomyocytes, the study by our group also detected autophagosomes within these podocytes and identified alterations in levels of LC3B (35), confirming loss of PRR disturbs autophagic flux.

In summary, in the 11 years since its discovery the contribution of PRR to the pathogenesis of cardiovascular disease and hypertension remains unclear. However, one clear and striking result from the above-mentioned conditional knockout studies in cardiomyocytes and podocytes is that the loss of PRR results in a disturbance in cellular autophagic flux and homeostasis (33–35). This is in addition to knockout models in other organisms where the loss of PRR disturbs cellular function and development. This is confirmed by the description of new tissue-specific PRR knockout model (ATP6AP2^lox/Y^; Hoxb7-Cre), which also has severe developmental effects (36). Taken together, the regulation of autophagy and cellular homeostasis is thus looking more likely to be the true cellular function of PRR and is the focus of this review.

## Autophagy

Autophagy is derived from the Greek names of *auto* “self” and *phagein* “to eat,” and describes the process by which the cell literally eats itself. This is an essential and evolutionary conserved process utilized by all cell types to maintain cell homeostasis. Under normal conditions, a basal level of autophagy is required to maintain protein quality control and remove damaged proteins, organelles, and lipids, which may otherwise harm normal cellular function (37). With aging, the rate of basal autophagy is thought to decline and it has been proposed that this reduction in autophagic flux results in the accumulation of damaged proteins and the induction of neurodegenerative and cardiovascular diseases (38, 39). The level of basal autophagy has been shown to vary between different cell types with terminally differentiated cell types, such as neurons, having very high levels of autophagic flux (40).

**Figure 1:**
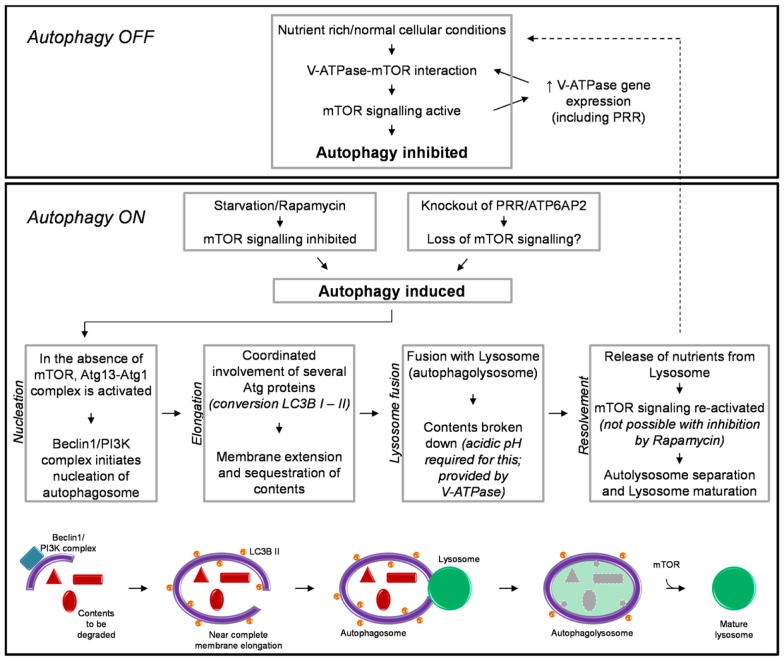
**A brief schematic of the regulatory processes involved in autophagy (see text for detail)**. Also shown is our hypothesized mechanism by which knockout of PRR/ATP6AP2 results in a loss of mTOR activity and the induction of autophagy.

Autophagy also has an essential role in response to conditions of cellular nutrient deprivation or starvation. Under these conditions, macroautophagy (hereafter referred to as autophagy) is induced as a way in which to replenish nutrients and prevent cell death, *via* the degradation of cellular proteins and organelles to generate amino acids. Due to its important role in maintaining cellular homeostasis the process of autophagy is extremely tightly regulated. This process involves multiple steps and the converging of several signaling pathways, requiring the coordinated action of literally hundreds of proteins. The specific details involved in the regulation, initiation, action, and resolvement of autophagy have been discussed extensively elsewhere (41, 42). For the purpose of this review, a brief summary is shown in Figure 1.

The induction of autophagy is controlled by an atypical serine kinase, the mammalian target of rapamycin (mTOR). Under normal conditions, where the cell is in a nutrient rich state or with a lack of stress signals, mTOR is active and inhibits autophagy. However, once in a nutrient-deprived or stressed state, the activity of mTOR is reduced/inhibited, and the process of autophagy is initiated.

Briefly, autophagy begins with the nucleation of the autophagosome membrane. Upon a decrease in mTOR activity, the autophagy-related protein (Atg) 13 is dephosphorylated, allowing it to form an activating complex with another “Atg” protein, Atg1. The assembly of this complex initiates the formation of the autophagosome membrane, driven by Beclin-1 and a phophoinositide-3 kinase (PI3K) complex (Figure 1). The membrane is then extended around the contents to be degraded. The extension of this membrane involves numerous other Atg proteins but the most important, due to its common use in histology and western blotting to detect autophagy, is the protein light-chain (LC) 3B (in yeast known as Atg8). LC3B is normally present in the cytoplasm (LC3B I) and it is only upon its post-translational modification, where a lipid group (phosphatidylethanolamine) is added (LC3B II) that it associates with the membrane of the autophagosome. This modification is commonly used as a marker for autophagy as the more hydrophobic LC3B II runs at a different molecular weight than LC3B I when analyzed by western blotting (43, 44). When the cellular contents to be degraded are completely enclosed by the autophagosome, the next step of autophagy involves the fusion of this body with a lysosome; thus forming an autophagolysosome. The lysosome is comprised of various proteases which require acidic pH for their activity. This pH is provided by the activity of the vacuolar H^+^-ATPase (V-ATPase; discussed further in the next section). After degradation of its contents and the release of amino acids, the autophagolysosome separates and autophagy is attenuated. Importantly, the separation and maturation of lysosomes back to their normal state has been shown to also be regulated by mTOR (Figure 1) (45). Thus an important self-regulatory negative feedback mechanism is established in which after the restoration of cellular nutrients, mTOR is reactivated and both inhibits the induction of further autophagy and is involved in the resolvement of cellular homeostasis, *via* the removal of autophagosomes from the cell and promoting the maturation of lysosomes and return to their normal morphology (45).

Modulation of autophagy regulatory networks can have many different effects, depending on the level at which the autophagy pathway is inhibited, the cell type and/or disease in question in which such a modulation is utilized. Rapamycin is a chemical derived from *S. hygroscopicus* and is a potent inhibitor of mTOR activity and thus is an inducer of autophagy (46). Incubation of a number of different cell types with rapamycin results in the induction of autophagy and eventual death, due to a failure of the cells to attenuate and resolve autophagy (47–51). However, in some situations this can be of benefit. Rapamycin is currently approved for clinical use to treat certain types of cancers such as breast, colorectal, and renal. Here, rapamycin potently inhibits cancer cell proliferation and is able to induce the death of cancerous cells and even inhibit angiogenesis (52). However rapamycin is not an effective drug for all clinical situations, as induction of autophagy can also have detrimental effects to the function of non-diseased cells (53). Thus for the use and further development of therapeutics such as this, a thorough understanding of the requirements of different cells for autophagy is crucial.

## The Vacuolar H^+^-ATPase and Its Role in Autophagy

The “discovery” of PRR was eventually realized not to be the discovery of a *novo* protein after all. It is now well established that PRR is actually *ATP6AP2*; a gene product identified as an accessory protein of the V-ATPase (54). PRR has been shown to co-localize and immunoprecipitate with the V-ATPase, indicating a functional association (54, 55). In zebrafish, the loss of PRR results in a loss in pigmentation phenotype which directly mimics that of loss of a V-ATPase subunit (27). Additionally, the phenotype of the cardiomyocytes-specific PRR knockout model was attributed by the authors to a loss of V-ATPase function (33).

The V-ATPase is an essential multi-subunit complex present in nearly all cell types. It is responsible for establishing and maintaining intracellular pH gradients. Thus, its importance in maintaining cellular homeostasis is considerable. These duties range from the acidification of the lysosome, endocytosis, and recycling of membrane proteins, secretion and processing of hormones such as insulin, and basic cellular trafficking including the fusion of vesicular membranes (29, 56, 57).

It has recently been shown that the V-ATPase is important for the above-described mTOR signaling pathway, involved in regulating autophagy (Figure 1) (58). Addition of specific inhibitors of V-ATPase to the culture media of HEK293 cells inhibited the activity of mTOR. Additionally, immunoprecipitation studies identified an interaction between V-ATPase subunits and the Rag-Regulator complex, which interacts with mTORC1 to form the active mTOR signaling complex. This study proposed a mechanism by which the V-ATPase is thus crucial in maintaining mTOR activity by sensing the nutrient state of the cell and modulating the interaction between mTORC1 and the Rag-Regulator complex (58).

An important regulatory network between mTOR and the expression of V-ATPase subunits has also been identified (59). In a cell line with a genetically abnormally increased activity of mTOR, the expression of V-ATPase subunits was also increased. Of note is that PRR/ATP6AP2 was one such subunit identified to have increased expression under these circumstances. It was therefore concluded that mTOR regulates the expression of V-ATPase subunits (59). Considering the previous study by Zoncu et al. (58), this results in the development of a positive feedback loop whereas V-ATPase subunits are essential for maintaining mTOR activity and *vice versa* (Figure 1).

## PRR: A Protein Important for Regulating Autophagy?

The best insight into what is the precise contribution of PRR to autophagy comes from analysis of the recent studies by Riediger et al. (35) and Oshima et al. (34). These studies both generated mice with PRR specifically deleted in podocytes; a specialized cell of the kidney. As mentioned briefly above, loss of PRR in this cell type resulted in animal mortality approximately 3 weeks after birth (Table 1) (34, 35). The cause of this severe phenotype was due to the animals developing nephritic syndrome and acute kidney injury; identified by proteinuria, glomerulosclerosis, and the accumulation of proteaceous casts in tubules. Upon inspection by transmission electron microscopy, the accumulation of vesicles was observed in addition to the presence of large autophagosomes, also after only 3 weeks. Additionally, an accumulation of LC3B was detected, indicating that the deletion of PRR in podocytes results in a disturbance in autophagic flux (34, 35). This mirrors what Kinouchi et al. observed in their cardiomyocyte-specific PRR knockout model, as described above (33). Eventually this disturbed autophagic flux led to podocyte death, as indicated by a decrease in Wilms tumor-1 signal (34, 35). This did not correlate with the activation of apoptotic pathways, indicating that the gross disturbance in autophagy was the main cause of cell loss (35).

**Table 1:** **Comparison of the time of onset of various parameters in PRR and autophagy-related podocyte-specific knockout mouse models**.

	(Pro)renin receptor (34, 35)	Atg5 (60)	mTOR (61)
Genotype	ATP6AP2^lox/Y^; Pod-Cre	ATG5^flox/flox^; Pod-Cre	mTOR^flox/del^; Pod-Cre
Mortality	3 weeks	No effect (mice live >24 months)	Not analyzed
Proteinuria (albumin/creatinine)	2 weeks	Mild at 8–12 months, severe at 20–24 months	3 weeks
Glomerulosclerosis	2 weeks	24 months	4 weeks
Proteinaceous casts in tubules	2 weeks	24 months	2–4 weeks
Podocyte number	Decreased at 2 weeks	Decreased at 22 months	Not analyzed
Podocin expression	Decreased at 3 weeks (not analyzed by Riediger et al.)	No change at 24 months	Decreased at 3 weeks
Podocyte foot effacement	2 weeks	24 months	3 weeks
Autophagosome formation within podocytes	2 weeks	8–12 months	2 weeks
Alteration in LC3B processing	Accumulation of LC3B positive cells (immunofluorescence)	Not analyzed	Increased LC3B II conversion (western blot)

Two separate conditional knockout studies in the podocyte have been generated to specifically investigate the role of autophagy in this cell type (Table 1). Atg5 is a protein important for the elongation of the autophagosome membrane and sequestration of contents (Figure 1). In contrast to PRR, conditional deletion of *ATG5* in podocytes resulted in severe kidney disease only after 24 months, with no animal mortality at this time (60). The presence of autophagosomes within Atg5 knockout podocytes was evident only after 8–10 months (Table 1). In this study, the authors proposed that loss of Atg5 results in a gradual decrease in the cells ability to remove unwanted and damaged cellular material (60). In this case, the late onset of disease can be attributed to the cells lacking a functioning *basal* autophagy and so, first a certain threshold of cellular stress must be reached (i.e., by aging) before the phenotype is evident.

In clear contrast to this is the study by Cina et al. who generated podocyte-specific *mTOR* knockout mice (61). Here, these mice developed proteinuria, glomerulosclerosis, and other hallmarks of acute kidney injury after only 3 weeks. Like both the *PRR* and *ATG5* knockout studies, autophagosomes were detected within the podocytes, however, more similar to the PRR knockout model, these were evident after only 2 weeks (Table 1). The authors propose in this study that the loss of mTOR in podocytes has a twofold effect (61). Firstly, loss of mTOR results in the induction of autophagy, as indicated by the presence of autophagosomes and accumulation of LC3B II. Secondly, the authors demonstrate that loss of mTOR results in a failure of negative feedback loops to stop the induction of further autophagy and resolve this process. Hence, they propose that the severe and acute nephritic syndrome in mTOR podocyte-specific knockout mice is due to the disruption of the autophagic cycle at two points: induction and resolvement (Figure 1).

What is striking is the similarity in the severity of the phenotype observed between the conditional knockout of PRR and mTOR in these podocyte studies (Table 1). This strongly suggests that PRR is important in mTOR function, either due to a specific interaction or indirectly *via* its association with the V-ATPase (58). In support of this concept is that ubiquitous mTOR knockout mouse models also have similarities to that of PRR, where mTOR knockout embryonic stem cells have limited proliferation resulting in early lethality (E5.5), indicating an essential role for mTOR in cellular development (62, 63). The comparison of future PRR and mTOR conditional knockout models in other cell types will give more insight into the molecular mechanism by which PRR is important for mTOR activity.

## Where to Next?

There is now clear evidence that PRR has an essential role in maintaining cellular homeostasis, specifically due to its involvement in autophagy. This will undoubtedly result in shift in research focus away from the contribution of PRR to cardiovascular disease, toward understanding its general role in the biology, homeostasis, proliferation, and development of all cell types.

It must be acknowledged that this paradigm change was initiated by the study by Cruciat et al. (29). This group essentially stumbled across PRR as being important in canonical Wnt signaling as part of their large research study to identify new genes of importance to this signaling pathway. In this study, they showed a clear link between the association of PRR with the V-ATPase and the activation of protein receptors important for the induction of Wnt signaling (29). It has also been established in *Drosophila* that PRR is important for another Wnt signaling pathway, the planar-cell polarity (PCP) pathway (30). More recently, this group has dissected the mechanism by which PRR is important for PCP signaling. Here, loss of PRR affected the co-localization and endocytosis of receptors important for PCP, and resulted in defects in the degradation of other receptors such as Notch and E-Cadherin (64). As discussed in this review, it also appears likely that PRR has an important role in the signaling pathways important for regulating autophagy. We therefore propose that PRR is essential for proteostasis, where the loss of this protein results in the disturbance of multiple signaling pathways, resulting in severe defects in cellular homeostasis. This could be due to its role in regulating autophagy, in which loss of PRR results in the disturbance of multiple signaling pathways, due to the induction of autophagy and lack of resolvement of this process, resulting in eventual cellular death. It is also possible, as with the study described by Cruciat et al. that PRR specifically interacts with proteins important for signal transduction. Of note is that the signaling pathways of Wnt and autophagy are closely intertwined (65). Deciding which of these two hypotheses is correct will be made easier once the precise molecular mechanism by which PRR contributes to V-ATPase activity is determined. This is a particularly important concept to understand in light of the fact that lower organisms which lack PRR (e.g., yeast) still have a functioning V-ATPase (33). The answers to these questions will lead to new insight into the regulatory pathways essential for maintaining cellular homeostasis.

## Conclusion

It is now clear that PRR has an important role in the regulation and maintenance of cellular homeostasis, most probably *via* signaling pathways important for autophagy. In consideration of this new information, it would be interesting to revisit the original studies describing the involvement of PRR with the RAS and regulation of blood pressure, to distinguish the role of V-ATPase function, and autophagy in these systems. More recent papers, which have identified a role for PRR in various signaling pathways, should also be carefully re-examined to deduce whether these effects are directly mediated by PRR, or indirectly by the gross disturbance of cellular homeostasis.

## Conflict of Interest Statement

The authors declare that the research was conducted in the absence of any commercial or financial relationships that could be construed as a potential conflict of interest.
